# Immune response and treatment targets of chronic hepatitis B virus infection: innate and adaptive immunity

**DOI:** 10.3389/fcimb.2023.1206720

**Published:** 2023-06-22

**Authors:** Peiyu Zheng, Yongqing Dou, Qinying Wang

**Affiliations:** ^1^ Department of Infectious Diseases, The First Hospital of Shanxi Medical University, Taiyuan, China; ^2^ Graduate School of Shanxi Medical University, Taiyuan, China

**Keywords:** chronic hepatitis B, innate immunity, adaptive immunity, immune tolerance, treatment

## Abstract

Chronic hepatitis B virus (HBV) infection is a major global public health risk that threatens human life and health, although the number of vaccinated people has increased. The clinical outcome of HBV infection depends on the complex interplay between viral replication and the host immune response. Innate immunity plays an important role in the early stages of the disease but retains no long-term immune memory. However, HBV evades detection by the host innate immune system through stealth. Therefore, adaptive immunity involving T and B cells is crucial for controlling and clearing HBV infections that lead to liver inflammation and damage. The persistence of HBV leads to immune tolerance owing to immune cell dysfunction, T cell exhaustion, and an increase in suppressor cells and cytokines. Although significant progress has been made in HBV treatment in recent years, the balance between immune tolerance, immune activation, inflammation, and fibrosis in chronic hepatitis B remains unknown, making a functional cure difficult to achieve. Therefore, this review focuses on the important cells involved in the innate and adaptive immunity of chronic hepatitis B that target the host immune system and identifies treatment strategies.

## 1 Introduction

Chronic hepatitis B virus (HBV) infection is a major public health concern. Persistent HBV infection leads to the development of liver fibrosis, cirrhosis, and hepatocellular carcinoma (HCC), which threaten human health. In 2019, approximately 296 million people suffered from chronic HBV infection, and 820,000 died from HBV-related diseases such as cirrhosis, hepatocellular carcinoma, and liver failure (GBD 2019 Hepatitis B Collaborators, 2022). In 2016, 86 million people in China were chronically infected with HBV (6.1% of the population). The natural history of chronic HBV infection can be divided into four stages: immune tolerance, immune activity, inactive carriers, and reactivation. HBV infection does not directly cause liver cell damage. The clinical outcome of infection depends on the complex interplay between HBV replication and host immune response. Currently, nucleotide analogs and interferons (IFNs) are used for the treatment of chronic hepatitis B (CHB). However, these drugs cannot completely eliminate covalently closed circular DNA (cccDNA), which plays an important role in persistent HBV infection and hepatitis B recurrence, resulting in failure to cure CHB. Therefore, research on the clinical cure for CHB has become a hot topic in recent years to achieve the goal of “eliminating viral hepatitis as a major public health threat by 2030,” as set by the World Health Organization (Chinese Society of Hepatology et al., 2022).

Chronic hepatitis B is primarily caused by a pathognomonic immune response. Host immune responses, including innate and adaptive responses, lead to liver inflammation and damage while fighting the virus. Although adaptive immunity is key to controlling and clearing HBV infection, the role of innate immunity cannot be ignored. Adaptive immunity depends on the activation signals and cytokines secreted by the innate immune system. HBsAb can bind to HBsAg to limit its spread and kill or phagocytose HBV-infected cells. HBcAg preferentially induces Th1 responses and activates cellular immune responses, whereas HBeAg promotes Th2 responses related to humoral and anti-inflammatory immunity (Khanam et al., 2021). Immune responses and cytokine stimulation can affect cccDNA maintenance, and immune-mediated elimination of cccDNA is an impressive new method. Hepatocytes eliminate cccDNA via noncytolytic clearance and killing of infected cells by CD8^+^T cells (Bhat and Kazim, 2022). The liver is an immunotolerant organ because it readily accepts allografts and does not require a major histocompatibility complex (MHC) match, which can lead to persistent infections and rapid cancer progression (Zheng and Tian, 2019). The duration of HBsAg exposure, rather than the quantity of HBsAg, is associated with the anti-HBV immune response. Therefore, strategies to restore anti-HBV immune responses should be considered for patients younger than 30 years (Le Bert et al., 2020a).

**Figure 1:**
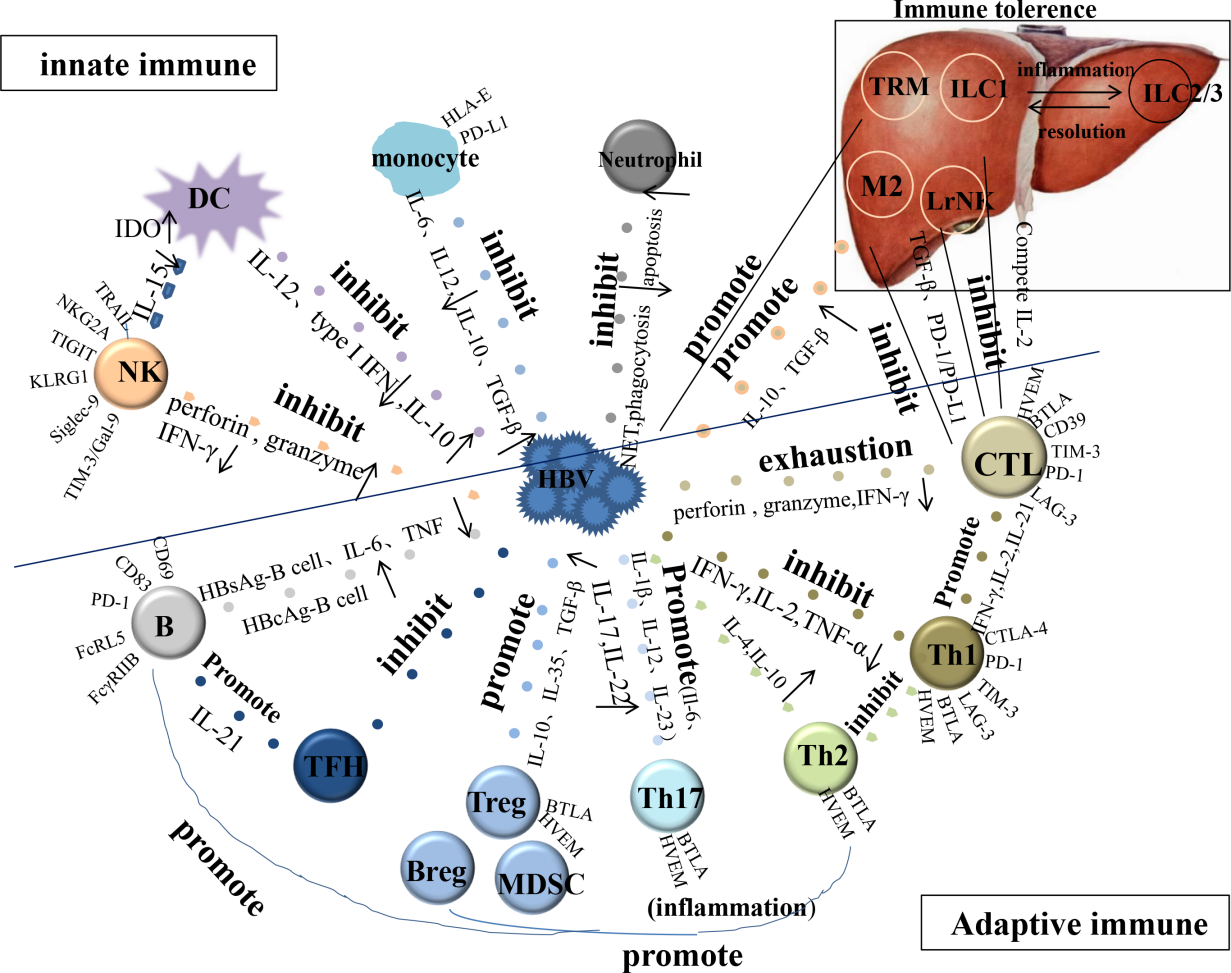
The function of important cells involved in CHB innate and adaptive immunity. HBV, hepatitis B viral; DC, dendritic cell; NK, natural killer cell; TFH, follicular helper T cell; CTL, cytotoxic T lymphocyte; LrNK, Liver-resident NK; M, Macrophage; TRM, tissue-resident memory T cell; IDO, indoleamine 2, 3-dioxygenase.

The functions of important cells involved in CHB innate and adaptive immunity (**Figure 1**), such as dendritic cells (DCs), natural killer (NK) cells, monocytes/macrophages, neutrophils, T and B cells, and suppressor cells have been reviewed to target the host immune system and identify treatment strategies.

## 2 Innate immunity cells in patients with chronic hepatitis B

Innate immunity plays an important role in the early stages of infection. The complementary system is an essential component of the innate immunity and contributes to the initial defense against viral infections by activating a cascade of sequential proteins. The classical and alternative complement pathways are triggered by antigen-antibody complexes and foreign surface molecules, respectively. The lectin pathway involves binding of microbial surface carbohydrate moieties to serum mannose binding lectin (MBL)/ficolin, thereby activating MBL-associated serine proteases (MASPs) and proteins. Each pathway converges during the formation of C3 convertase, which activates the downstream complement factors to form a membrane attack complex (MAC). MAC form pores in the lipid membranes of the pathogens and infected cells, resulting in osmotic lysis. However, HBV can integrate host complement regulatory proteins into its outer membrane, thereby escaping host complement attacks. Moreover, patients with high HBV DNA levels have a higher rate of bacterial infections (Agrawal et al., 2017).

C1q is the initiator and control protein of the classical pathway, and ApoE affects complement activation by inhibiting C1q activity. C1q-ApoE complexes are rare or absent in the normal liver. However, immune cells infiltrate the liver to form a large number of C1q-ApoE complexes in patients with CHB, which inhibit the activation of the classical pathway (Habenicht et al., 2022). C2 enhances the antiviral effect of IFN and inhibits HBV replication by inhibiting the p38-MAPK signaling pathway. HBV inhibits C2 expression by increasing the expression of Sp1 and reducing histone deacetylase 4 expression, thereby promoting HBV persistence (Ning et al., 2020). The expression levels of C3 and C4 in patients with CHB were significantly lower than those in healthy controls, and the decrease in C3 levels is consistent with the severity of HBV-related diseases (Zhu et al., 2018). C4a inhibits the secretion of HBV DNA, and C4a levels in patients with CHB were lower than those in healthy controls. Supplementation with low levels of C4a may help treat HBV infections (Song et al., 2015). C5a can upregulate the expression of α-sma, the production of hyaluronic acid and type IV collagen, and inhibit the apoptosis of hepatic stellate cells (HSCs), which is positively correlated with the severity of liver fibrosis (Xu et al., 2013). HBV reduces C9 activity in a dose-dependent manner by blocking USF-1, which is essential for C9 transcription, whereas the expression of other MAC components (C5b,6-8) remains unchanged, thus blocking the formation of MAC (Baidya et al., 2022). CFB is a key molecule in the alternative pathway that plays an important role in the amplification cascade of complement activation. It also inhibits HBV replication and HBV can inhibit CFB expression (Chen et al., 2020). Complement factor H (CFH) inhibits the formation of C3 converase (C3bBb) via an alternative pathway, and HBx induces miR-146a to downregulate CFH and promote liver inflammation (Li et al., 2015a). CD59, an important complement system inhibitor, prevents MAC formation. HBV promotes the translocation of CD59 to the nucleus, and the downregulation of CD59 leads to increased MAC deposition, promotes complement-mediated lysis, and aggravates liver injury. Inhibition of the HBV-CD59 interaction may prevent the occurrence or development of liver inflammation (Liu et al., 2013).

MBL recognizes HBsAg through its carbohydrate-recognition domain to bind HBsAg in a dose-dependent manner; this binding is calcium-dependent, and mannose is inhibited. Moreover, MBL enhanced C4 deposition on HBsAg, indicating that MBL is involved in HBV clearance by activating complement on the HBsAg-MBL complex through the lectin pathway (Chong et al., 2005). The frequency of haplotypes associated with low serum MBL is significantly increased in chronic HBV infection; low MBL levels lead to defective complement activation, poor clearance of immune complexes, and subsequent deposition in the liver, leading to the persistence of HBV infection and disease progression (Filho et al., 2010). MBL polymorphisms affect the biological activity of MBLs, and defective interactions with MASPs, carbohydrate recognition domains, and their targets lead to uncertain activation of complementary systems (Moura et al., 2017). The results of studies on the potential association between MBL polymorphisms and CHB have been inconsistent. A meta-analysis showed that polymorphisms in the MBL promoter and exon 1 are significantly associated with HBV infection, and Allele D is associated with HBV persistence (Qie et al., 2019). Ficolin-2 levels are positively correlated with the degree of liver inflammation in patients with CHB. Patients with higher ficolin-2 concentrations are prone to HBeAg seroconversion, resulting in better outcomes (Chen et al., 2015). Ficolin-2 promoter polymorphism is associated with ficolin-2 concentrations, the AGGG haplotype contributes to the prevention of HBV infection and liver cancer, and AAAG is associated with higher serum ficolin-2 levels (Hoang et al., 2011).

Generally, pattern recognition receptors (PRRs) on immune cells recognize pathogen-associated molecular patterns (PAMPs), produce IFN, and express IFN-stimulated genes, thereby limiting the replication and spread of viruses (Megahed et al., 2020). However, HBV, a “stealth” virus, neither induces nor interferes with innate immunity in patients with HBV infection (Suslov et al., 2018). Thus, the autonomous innate immune system in the cells remains intact. This may be a strategy by which HBV evades detection by the host innate immune system. Therefore, compounds capable of activating anti-HBV innate immunity have been evaluated as novel therapeutic interventions for CHB (Suslov et al., 2018). Moreover, we analyzed the characteristics of cellular involvement in the innate immunity of patients with CHB.

### 2.1 Dendritic cells

Dendritic cells (DCs) are the most potent antigen-presenting cells that can directly activate naïve T cells and initiate adaptive immune responses. According to their origin, they are divided into CD11c+ myeloid dendritic cells (MDCs) and CD123+ plasmacytoid dendritic cells (PDCs). Immature DCs express low levels of MHC II, costimulatory, and adhesion molecules; therefore, their ability to stimulate an immune response is weak. After antigen and inflammatory stimulation, the expression of these molecules increases, activating T cells to initiate an adaptive immune response (Sabado et al., 2017). Toll-like receptors 9 (TLR9) are highly expressed in PDCs and activate the NF-κB pathway to produce type I IFN and other proinflammatory cytokines associated with antiviral immunity (Durai and Murphy, 2016; Tsukamoto et al., 2020). MyD88 is a prerequisite for IFN promoter activation. Furthermore, polyamines (produced by DCs or arginase (Arg)1^+^MDSCs) convert DCs to indoleamine 2, 3-dioxygenase (IDO)-dependent immunosuppressive phenotypes by activating Src kinase and secreting IL-10 and TGF-β (Mondanelli et al., 2017; Salah et al., 2021).

After 24 h of exposure to HBV, the maturation of DCs was inhibited and costimulatory molecules and activation markers (CD83, CD80, CD40, and CD25) were damaged in a time- and dose-dependent manner, weakening the production of IL-12 and the Th1 cell immune response. Antiviral treatment restores DC function and significantly increases IL-12 levels (De Pasquale et al., 2021). However, Li et al. showed that although the maturation of DCs increased after antiviral treatment, the maturation level of DCs in patients with high HBsAg levels before treatment was still lower than that in patients with low HBsAg levels (Li et al., 2020). Therefore, high HBsAg levels may exert long-term inhibitory effects on DCs. HBsAg, HBeAg, and IFN-inducible protein 10 (IP-10) may contribute to PDC dysfunction. The TLR9, MyD88, and NF-κB in PDCs are significantly downregulated, type I IFN level is decreased, and immune function is defective (Zhu et al., 2021). The increased frequency of PDCs and high expression of CD86 after treatment help achieve a functional cure (Cao et al., 2022). HBV also induces DCs to produce IL-10, which in turn mediates immune tolerance. Blocking IL-10 restored DC maturation and stimulated NK cells. Lymphocytes activated by mature DCs significantly inhibited the synthesis and secretion of cccDNA mediated by cytokines, while IL-2 and IFN-γ were increased, and IL-10 was decreased, which activated the proliferation of HBV-specific T cells and cleared HBV (Shen et al., 2015). Therefore, enhancing the activation and antigen-presenting activity of DCs can promote the inhibition of HBV proliferation by the immune system (Liu et al., 2015).

To understand the molecular mechanisms underlying DC dysfunction in chronic HBV infection, Singh et al. analyzed the miRNA-mRNA levels in DCs at different stages of chronic HBV infection (Singh et al., 2021). miR-615-3p, miR-2278, and miR-3681-3p were downregulated in the immune active stage, which upregulated key immune regulatory genes in the MHC-I pathway, such as calnexin (CANX), calreticulin (CALR), HSP70, HSP90, HLA-A, and HLA-B, stimulating the function of CD4^+^ and CD8^+^ T cells and NK cells. HLA expression is indispensable for the immune response of CD8^+^ T cells, and its imbalance may be related to the progression and severity of liver diseases (Singh et al., 2018). Compared to those in the immune active stage, miR-152-3p, miR-3613-3p, the above immunomodulator genes (except CALR), and the proteasome activation subunit PA28 were decreased in the inactive carrier stage, thereby downregulating the MHC-I pathway. miR-152 is frequently downregulated in HBV-related liver cancer tissues. Therefore, HCC-related markers should be monitored regularly (Huang et al., 2010). The reactivation stage showed lower CALR and HSP70 and higher CANX, HSP90, and HLA-A levels than the inactive carrier stage, and the MHC-I pathway was activated. Moreover, the miRNA–mRNA network in DCs is associated with an impaired immune response during chronic HBV infection.

DC–NK cell interactions are also influenced by persistent HBV infection because of DC dysfunction, which is beneficial for DC maturation and NK cell activation. HBV impairs the ability of DCs to induce proliferation and maintain NK cell activity by secreting IL-15 to control viral infections. The mechanism may involve the overexpression of the IDO protein and mRNA, which is a tolerogenic enzyme that inhibits the proliferation of NK and T cells (Pietra et al., 2012). IDO inhibitors restore NK cell proliferation; IL-12 promotes the production of IFN-γ by NK cells and the differentiation of T cells into Th1 cells and upregulates antigen presentation and costimulatory molecules on mature DCs to activate T cells and enhance adaptive immune responses. Immune proteasome (IP) is an important component of antigen-presenting DCs; it is generated by the proteasome and IFN-γ-induced subunits, such as LMP2, LMP7, and LMP10, and is selectively expressed in the activated DCs to increase the production of antigen peptides. Normally, NK cells secrete IFN-γ, which induces the expression of the LMP2 and LMP7 subunits (Hensley et al., 2010). However, LMP2 and LMP7 are defective, and NK cells cannot induce the expression of LMP2 and LMP7 subunits in the presence of HBV, which may be related to increased IL-10 levels and decreased production of IFN-γ by NK cells. NK cells play a regulatory role by selectively editing DCs and recognizing low levels of HLA I expressed on immature DCs and killing them as they cannot optimally initiate T cell responses, leading to immune tolerance. HBV can upregulate HLA I molecules in DCs, protecting them from the killing effect of NK cells. Simultaneously, PDCs evade immunity by impairing the killing activity of NK cells via OX40L/IFN-dependent pathways. HBV-stimulated DCs show decreased CCR6 and increased CCR7 expression. It can promote the migration of DCs to the lymph nodes and lead to immune tolerance (De Pasquale et al., 2021). Disruption of DC–NK cell interactions reduces the immune control of HBV and leads to chronic infection (Martinet et al., 2012).

### 2.2 Natural killer cells

NK cells, which account for about 31% of the total number of intrahepatic lymphocytes (Zheng et al., 2018), can directly kill infected cells through intercellular contacts (perforin, granzyme, granulysin or Fas/FasL, and TNF/TNF-related apoptosis-inducing ligand (TRAIL)) or by secreting antiviral cytokines (IFN-γ or TNF-α) to achieve antiviral activity (Ali et al., 2019). NK cell activity is regulated by activating receptors (KIR, NKG2D, NCR (NKp46, NKp30, and NKp44)) and inhibitory receptors (IRs) (KIR and NKG2A) (El-Deeb et al., 2019). CD69 is an activation marker of NK cells (Peng and Tian, 2018). Moreover, NK cells can be divided into two subsets: CD56^bright^ CD16^-^ (CD56^bright^) and CD56^dim^ CD16^+^ (CD56^dim^). The former has a strong ability to produce cytokines and is highly expressed in the liver (Highton et al., 2021). The latter has a strong cytotoxic capacity and is predominant in the peripheral blood. CD56^bright^ cells are the precursors of CD56^dim^ cells (Fisicaro et al., 2019). STAT1 can regulate NK cell maturation, cytokine secretion, and cytotoxicity and plays an important role in host antiviral innate immunity (Gotthardt et al., 2019; Liu et al., 2021b).

HBV components can be transferred to NK cells, causing them to be biased toward cell-killing activity, preferentially accumulating in the liver and exacerbating liver damage without viral clearance (Zheng et al., 2015). HBV DNA, HBV RNA, and HBsAg are present in the serum exosomes of patients with CHB and are transferred to hepatocytes to induce infection and promote HBV transmission. Meanwhile, TGF-β can promote the uptake of exosomes by NK cells, resulting in NK cell dysfunction and reduced survival rate (Yang et al., 2017). TRAIL is highly expressed in activated CD56^bright^ NK cells and is a cytotoxic molecule that induces hepatocyte apoptosis. CD56^bright^ NK cells express CXCR3, which binds to ligands (including CXCL9, CXCL10, and CXCL11), and the frequency and activation of TRAIL- and CXCR3-related chemokines increases with severe liver injury (Falschlehner et al., 2009). TRAIL^+^CD56^bright^ NK cells migrate to the liver in the presence of CXCR3 receptor ligands, leading to liver injury and fibrosis. NK cells accelerate the progression of CHB by enhancing TRAIL and perforin-dependent cytotoxic activity, and by reducing the secretion of IFN-γ to promote liver injury and inhibit antiviral immunity (Ghosh et al., 2016). TRAIL-R2 is also upregulated in HBV-specific CD8^+^ T cells. Therefore, NK cells mediate the depletion of HBV-specific T cells via TRAIL, which is accompanied by caspase-8 activation (Peppa et al., 2013; Lam and Lanier, 2017). HBV induces NK cell immune tolerance by downregulating the expression of RIG-I and inhibiting the NF-κB and p38 signaling pathways. They display a transcriptional signature resembling that of exhausted T cells, and the transcription factor TOX drives NK cell dysfunction, depending on the calcium-associated transcription factor NFAT. Therefore, it can be considered as a potential therapeutic target (Marotel et al., 2021).

Chronic HBV infection can affect NK cell subsets, leading to the transformation of NK cells into CD56^-^NK cells in the peripheral blood, and its frequency is positively correlated with HBV DNA (Wijaya et al., 2021). The expression levels of KIRs, CD57, and KLRG1 were higher in CD56^-^ NK cells compared to immature CD56^bright^ NK cells, but lower than in mature CD56^dim^ NK cells. NKP46 and NKG2D expression was significantly decreased, whereas NKG2A and TIM-3 expression was significantly increased (Sivori et al., 2019). The cytotoxic molecules (granzyme B, perforin, and granulysin) and antiviral cytokines (IFN-γ) were low (Judge et al., 2020), and STAT1 expression was downregulated. HBeAg promotes the production of IL-10 by Tregs, leading to increased NKG2A and ILT2 expression in CD56^dim^ NK cells and cell dysfunction (Villa-Álvarez et al., 2017). Blocking NKG2A and ILT2 enhances the production of IFN-γ and TNF by CD56^dim^ NK cells (Ma et al., 2020; Zhang et al., 2022). TGF-β1 is a negative regulator of IFN-γ production, and its concentration is positively correlated with the percentage of ILT2^+^CD56^dim^ NK cells (Regis et al., 2020). After telbivudine treatment, the number of CD56^bright^ NK cells and IFN-γ were significantly increased, and the function of CD56^bright^ NK cells was restored by upregulating the expression of IL-15 and NKG2D (Chen et al., 2017). IL-15 enhances NKG2D-mediated signaling by increasing the levels of NKG2D and expression of the intracellular adapter DAP10 through ERK phosphorylation. Activation of STAT1 can directly regulate the transcription of NKG2D, enhance the killing function of NK cells, and inhibit their apoptosis, thereby enhancing the immune response (Huang et al., 2022b). These targets modulate NK cells dysfunction in various ways. APOBEC3s are a family of cytidine deaminases that degrade cccDNA via their DNA deamination activity in response to stimulation by different cytokines. APOBEC3A (A3A) and APOBEC3B (A3B) are essential for inducing cccDNA degradation. CD56^bright^ NK cells contribute to the loss of cccDNA by activating A3A and A3B expression in infected hepatocytes via TNF-α and IFN-γ (Shi et al., 2018a). However, the frequency of the A3B deletion allele is higher in persistent HBV carriers compared to healthy controls (Zhang et al., 2013).

#### 2.2.1 Other inhibitory receptors on natural killer cells

These other IRs are also expressed in NK cells and play different roles in chronic HBV infection.

The T cell immunoglobulin and ITIM domain (TIGIT) pathway can reduce cytotoxicity, production of IFN-γ, and immune response of NK cells and increase the apoptosis rate of NK cells (Wang et al., 2015). In the immune-active stage, TIGIT expression is low and CD69 and CD107a expression is high, increasing cytotoxic activity and decreasing the apoptosis rate to induce severe liver injury. TIGIT expression increases during the inactive carrier stage and exerts protective effects against severe liver injury (Wang et al., 2021).

Killer cell lectin-like receptor G1(KLRG1) is an IR of the C-type lectin-like family. KLRG1-expressing cells have low proliferative capacity but retain their effector functions. The proportion of KLRG1^+^NK cells in CHB is increased with high cytotoxicity and IFN-γ levels; targets activate HSCs in a TRAIL-dependent manner to inhibit liver fibrosis. HSC-derived osteopontin activates KLRG1^+^NK cells. Thus, KLRG1^+^NK cells play an anti-fibrotic role in chronic HBV infection (Wijaya et al., 2021).

Siglec-9 is an inhibitory immunomodulatory factor that is selectively expressed in CD56^dim^ NK cells and defines a subset of NK cells with a mature phenotype and enhanced chemotactic potential (Jandus et al., 2014). The decrease in Siglec-9^+^ NK cells in patients with CHB is negatively correlated with HBeAg and HBV DNA levels. Siglec-9 ligand expression is increased in the liver biopsy tissues of patients infected with HBV or in hepatocyte cell lines stimulated with cytokines (IL-6 or TGF-β). This suggests that Siglec-9 contributes to the persistence of HBV infection, leading to chronic infection (Zhao et al., 2018).

Galectin-9 (Gal-9), a natural ligand of TIM-3, plays an important role in immune regulation by interacting with TIM-3 to induce apoptosis or the inhibition of effector functions (Okoye et al., 2020). The expression of Gal-9 in circulating NK cells of patients with CHB is high and associated with NK cell dysfunction (IFN-γ, TNF-α, CD107a, granzyme B, and perforin). NK cells lead to CD8^+^ T cell dysfunction and increased apoptosis via the Gal-9/Tim-3 pathway. Blocking Gal-9/Tim-3 restored the antiviral activity of CD8^+^ T cells (Liu et al., 2022c).

#### 2.2.2 Natural killer cell subsets

Liver-resident NK (LrNK) cells account for nearly half the total number of liver NK cells, most of which are CD56^bright^ cells (Peng et al., 2016; Male, 2017). The liver microenvironment and TGF-β are essential for maintaining the unique Eomes^hi^Tbet^lo^ phenotype in LrNK cells. CXCR6 expression is important for liver residence because of the expression of its ligand (CXCL16) in the hepatic sinusoid. LrNK cells are characterized by a high expression of negative regulatory genes encoding LAG3, PD-L2, TRAIL, PD-L1, and CD39 (Harmon et al., 2019), which inhibit IFN-γ expression. NK cells preferentially express cytotoxic effector genes encoding granzyme A, CD107a (LAMP1), perforin, and granzyme K. LrNK cells directly inhibit antiviral T cell response through PD-1/PD-L1. The blockade of PD-L1 restores antiviral T cell function and promotes the proliferation of T cells *in vitro* (Zhou et al., 2019).

Group 1 innate lymphoid cells (ILCs), including NK cells and ILC1 cells, express the transcription factor T-bet and produce IFN-γ. NK cells are cytotoxic and circulate in the blood, whereas ILC1s have little cytotoxic activity and are predominantly tissue-resident (Vivier et al., 2018). The recognition of hepatocyte antigens by CD8^+^ T cells triggers an increase in liver NK cells and ILC1s. Group 1 ILCs move along hepatic sinusoids and interact with HBV-specific CD8+T cells, suppressing CD8+ T cell proliferation by competing for local IL-2 but not inducing apoptosis. Thus, group 1 ILCs may limit the efficacy of IL-2 therapy, and depleting group 1 ILCs or impairing their ability to sense IL-2 would increase the number of HBV-specific CD8^+^ T cells in the liver but may enhance T cell-mediated organ damage (Spits et al., 2016). ILC2s and ILC3s transdifferentiate into ILC1s under proinflammatory conditions while maintaining the potential to revert once inflammation is resolved (Fumagalli et al., 2022).

### 2.3 Monocytes/macrophages

According to the expression of CD14 and CD16, monocytes can be divided into classical, intermediate, and nonclassical subsets. Classical monocytes are the most abundant subset and play an important role in host proinflammatory defense. Intermediate monocytes are involved in antigen presentation. The main functions of non-classical monocytes are immune surveillance and pro-inflammatory cytokine secretion (Yuan et al., 2017). Monocytes are transported to infection or inflammation sites and differentiate into M1 macrophages with a pro-inflammatory phenotype or M2 macrophages with an anti-inflammatory phenotype, depending on the microenvironment.

Classical monocytes were significantly decreased, whereas intermediate and nonclassical monocytes were increased in patients with CHB. Expression of TLR-2, TLR-4, and TLR-9 were decreased, whereas those of PD-L1 and HLA-E were increased. HBsAg induces monocyte immunosuppression through the MyD88/NFκB signaling pathway. Monocytes predispose CD4^+^ T cells to Tregs and Th2 cells and produce fewer pro-inflammatory cytokines (IL-12, TNF-α, and IL-6) and anti-inflammatory cytokines (IL-10 and TGF-β). HBsAg and IL-4 lead to defects in phagocytosis and the oxidative response of monocytes. β-catenin promotes the expression of CCR2 and recruits monocytes to the liver. One year of tenofovir treatment did not restore monocyte function to normal or reduce serum HBsAg or IL-4 levels (Dey et al., 2022). Targeting β-catenin in monocytes or reducing HBsAg and IL-4 levels may restore monocyte function and promote viral clearance.

Kupffer cells (KCs) are macrophages that reside in the liver and play a dual role in the immune response against HBV infection. The number of KCs in patients with CHB is increased, which activates the NF-κB pathway and increases the secretion of cytokines (IL-6, IL-1, and TNF-α), thereby promoting antiviral immunity. However, immune tolerance of KCs in the liver is dominant, leading to persistent HBV infection. KCs differentiate into M2 macrophages and upregulate PD-L1 and Gal-9 expression in the presence of HBeAg. HBcAg increases TLR-2 on the surface of KCs and produces IL-10, thereby promoting CD8^+^ T cell exhaustion (Li et al., 2015b; Li et al., 2015b; Liu et al., 2018). KCs promote the development of Tregs and inactivate follicular helper T cells (TFHs) and B cells by secreting IL-10, leading to the blockage of germinal center (GC) formation and HBsAb. KCs produce TGF-β, which activates HSCs to secrete extracellular matrix (ECM) proteins and induces liver cirrhosis (Li et al., 2012). Fas on the surface of hepatocytes bind to FasL secreted by KCs to promote liver inflammation and tissue damage, and Fas/FasL-mediated lymphocyte apoptosis induces immune tolerance. Activation of the TLR4 signaling pathway induces CD11b^+^ myeloid cells to differentiate into neutrophils and the M1 inflammatory phenotype, reducing intrahepatic KCs and promoting the immune response and clearance of HBV (Wang et al., 2022). IL-2 can promote KC subsets to cross-present antigens and HBV-specific naïve CD8^+^ T cells can recognize hepatocyte antigens, thereby improving the antiviral function of T cells (De Simone et al., 2021). The stimulator of interferon genes (STING) is a key adapter protein in DNA-induced innate immune activation (Li et al., 2022b). Activation of STING signaling in KCs increases the hepatic expression of interferon-inducible protein 16 (IFI 16), which recognizes and binds to cccDNA in the hepatocyte nucleus and promotes the heterochromatin of cccDNA by inducing a type I IFN response and targeting the interferon-stimulated response element in cccDNA, inhibiting cccDNA transcription and leading to functional silencing. Therefore, HBV can down-regulate the expression of IFI16 in the hepatocytes (Yang et al., 2020).

### 2.4 Neutrophils

Neutrophils fight viruses primarily through reactive oxygen species (ROS), phagocytosis, degranulation, and the formation of neutrophil extracellular traps (NETs). IL-12 produced by neutrophils stimulates NK cells to secrete IFN and both promote Th1 differentiation, thereby enhancing cytotoxic T cell activity. HBV inhibits the release of NETs through ROS-dependent ERK and p38MAPK and cannot effectively capture and kill viruses, whereas HBcAg and HBeAg enhance mTOR to reduce the autophagy of neutrophils and inhibit neutrophil activation and migration to the infection site, leading to T cell dysfunction and persistent HBV infection. IL-17 secreted by Th17 cells can activate neutrophils, which are associated with liver injury in CHB. NK cells may induce neutrophil apoptosis via NKp46- and Fas-dependent mechanisms, thereby limiting inflammation (Leu et al., 2014; Hu et al., 2019).

## 3 Adaptive immune cells in patients with chronic hepatitis B

Once HBV evades innate immunity, these innate immune cells activate adaptive immunity to clear the virus. Adaptive immunity includes cellular and humoral immunity. Moreover, HBV-specific T cells are required for HBV clearance.

### 3.1 Cellular immunity

CD4^+^ T cells promote antigen-presenting cells (APCs) activation through CD40L-CD40 interactions and cytokines. CD8^+^ T cells encounter antigens on activated APCs, and costimulation with CD27 on CD8^+^ T cells and CD70 on DCs is necessary to expand and differentiate into cytotoxic T lymphocytes (CTLs). Differentiation involves transcriptional and epigenetic changes and is regulated by transcription factors. T-bet and Eome determine the identity of CTLs, where T-bet promotes a transient effector state, and Eome promotes a memory precursor state. ID2 and Blimp-1 contribute to the transient terminally differentiated effector CTLs, whereas ID3 and Bcl6 contribute to the generation and maintenance of memory CTLs. CD4^+^ T cells (Ahrends et al., 2017) promote CD8^+^ T cell differentiation into effector and memory cells, downregulate co-inhibitory receptors, increase cell migration, express chemokine receptors to reach specific tissues and invade the corresponding tissues through matrix metalloproteinases. CTLs infiltrate at the inflammation site and produce inflammatory cytokines (IFN-γ and TNF-α) and cytotoxic molecules (granzyme B and perforin) to clear pathogens. HBV-specific CD8^+^ T cells are regulated by a balance between PD-1-mediated inhibitory and stimulatory signals from activated DCs (Isogawa et al., 2013). In chronic HBV infection, the key component of IP in APCs, 26S proteasome subunit, is downregulated. Functional decline in the ubiquitin-proteasome pathway is associated with immune response dysfunction (Schurich et al., 2016). Endoplasmic reticulum aminopeptidase 1 (ERAP1) trims HBcAg-specific peptides and binds to HLA-C∗04:01. ERAP1 promotes CD8^+^ T cell-specific antiviral immune responses by presenting antigen peptides via major histocompatibility complex I molecules (Liu et al., 2022b). Hepatocytes are the natural targets of HBV, which leads to the activation and proliferation of CD8^+^ T cells; however, these cells do not differentiate into effector cells. Antigen recognition in hepatocytes initiates a defective differentiation program with altered transcription, and ultimately, dysregulation of the T cell phenotype (Bénéchet et al., 2019). The frequency of core-specific CD8^+^ T cells is high, and the absence of envelope-specific CD8^+^ T cells may be highly reactive to the abundant HBsAg. Polymerase-specific CD8^+^ T cells exhibit their original phenotype. High expression of CD38, KLRG1, and Eomes and low expression of T-bet and CD127 indicate greater exhaustion of T cells, which is associated with an imbalance in TCF1/BCL2 expression (Schuch et al., 2019). The mechanisms of T cell exhaustion and the expression of IRs will be discussed in further detail.

#### 3.1.1 T Cell exhaustion

##### 3.1.1.1 Mechanism of T cell exhaustion

Long-term exposure to HBV antigens results in CD8^+^ T cell effector dysfunction, known as exhaustion, and even apoptotic deletion in severe cases. BIM-mediated apoptosis may lead to the exhaustion of CD8^+^ T cells (Ye et al., 2015). The gradual emergence of highly dysfunctional CD8^+^ T cells (Beltra et al., 2020) to prevent excessive immunopathology is facilitated by Eomes and T-bet while also contributing to chronic persistent infection. IR and cytokine levels vary among the different subsets of CD8^+^ T cells. Slamf6^+^ progenitor cells produce two CD8^+^ T terminally differentiated effector cell subsets: CX3CR1^+^ cells and Pdcd1^+^ cells (encoding PD-1). CX3CR1^+^ cells exhibit cytotoxicity, which limits viral replication. In contrast, Pdcd1^+^ cells were dysfunctional, with reduced cytokine production and cytolytic activity. IL-21 derived from CD4^+^ T cells promotes the differentiation of Ly108^+^ (encoded by Slamf6^+^) precursor cells into CX3CR1^+^ cells, while restricting their differentiation into Pdcd1^+^ subsets. The precursor Tcf-1^+^ (CD101^-^TIM3^-^), transient (CD101^-^TIM3^+^), and exhaustion (CD101^+^TIM3^+^) subsets in the study (Hudson et al., 2019) were similar to the Ly108^+^, CX3CR1^+^, and Pdcd1^+^ subsets in the above study.

Stem cell-like Tcf-1^+^ cells maintain T cell responses during chronic infection and generate effector T cells during PD-1 immune checkpoint blockade. The increased Tcf-1^+^ cells improve patient survival (Zander et al., 2019), and the TGF-β-mTOR axis plays a key role in this process (Kallies et al., 2020). The transcription factors, BACH2 and BATF, regulate early precursor and effector T cells in contrasting ways (Gabriel et al., 2021). NFATC1, IRF4, and NR4A are also highly expressed in exhausted T (Tex) cells. Preferential NFAT activation drives NR4As and TOX expression. TOX is indispensable for the formation of Tex cells, depending on the antigen dose at initial expression but not affinity (Utzschneider et al., 2020). TOX induces the exhaustion of genes, such as those encoding IR (PDCD-1, LAG-3, and CTLA-4) and transcription factors (NR4A2, IKZF3, TOX2, and BHLHE41), while inhibiting memory-related genes (CCR7, IL7r, and Sell). TOX is induced by calcineurin and NFAT2. Once the TOX-dependent Tex cell program is established, it is converted into a calcineurin-independent program. TOX is central to the epigenetic and transcriptional programs that coordinate Tex cell development and support long-term survival and maintenance (Alfei et al., 2019). Interference with the expression or activity of TOX can regulate T cell differentiation and increase effector function, which may be a strategy for immunotherapy (Khan et al., 2019; Seo et al., 2019).

Cytokines regulate acquisition and maintenance of T cell exhaustion. IL-10 acts on T cells directly through STAT-3 or indirectly by regulating APCs to promote T cell exhaustion and viral persistence (McLane et al., 2019). TGF-β and Smad2, characteristic of Tex cells, promote the differentiation of terminal Tex cells and inhibit mTOR signaling (Kurachi, 2019). IL-21 promotes the expression of the basic leucine transcription factor (BATF) involved in initiating or maintaining HBV-specific CD8^+^ cell effector function by promoting the proliferation and production of IFN-γ, granzyme B, and CD107a and decreasing PD-1 and TIM-3 production (Tang et al., 2019). IL-2 promotes the proliferation of CD8^+^ T cells by activating the mTOR pathway to restore dysfunctional CD8^+^ T cells caused by hepatocyte priming (Liu et al., 2022b).

During metabolism, T cell effector functions are mainly supplied by the energy generated by glycolysis and oxidative phosphorylation (OXPHOS). The oxidative metabolism of activated T cells is maintained by the mTORC1-CD28 signaling pathway and the transcription factor c-Myc, which promotes glucose and amino acid uptake by increasing the expression of GLUT1 and CD98 (Scharping et al., 2021). Early HBV-specific CD8^+^ T cell exhaustion downregulates glycolysis and OXPHOS associated with mitochondrial depolarization, thereby affecting the proliferation and effector functions of T cells. There are extensive mitochondrial genetic abnormalities and formation of non-functional giant mitochondria, including genes associated with electron transport, core mitochondrial protein synthesis, transmitochondrial membrane transport and metabolism, and changes in mitochondrial mass and membrane potential (Isogawa et al., 2013). IL-12 reduces the percentage of depolarized mitochondria, restores mitochondrial function, and reverses the dependence of T cells on glycolysis to achieve effector function. However, sufficient amounts are not produced by patients with CHB. PD-1 reduces mTOR-mediated expression of GLUT1 and glutamine transporters and activates mitochondrial lipid metabolism, leading to metabolic reprogramming toward a memory-like phenotype. T cell activation not only depends on mitochondrial biogenesis and polarization but also on balanced ROS production (Schurich et al., 2016). High ROS levels in Tex cells lead to DNA damage and promote T cell death. Mitochondrial antioxidant treatment restores mitochondrial depolarization and elevates ROS levels in T cells, resulting in a significant increase in cell survival and antiviral activity (Fisicaro et al., 2017).

**Figure 2:**
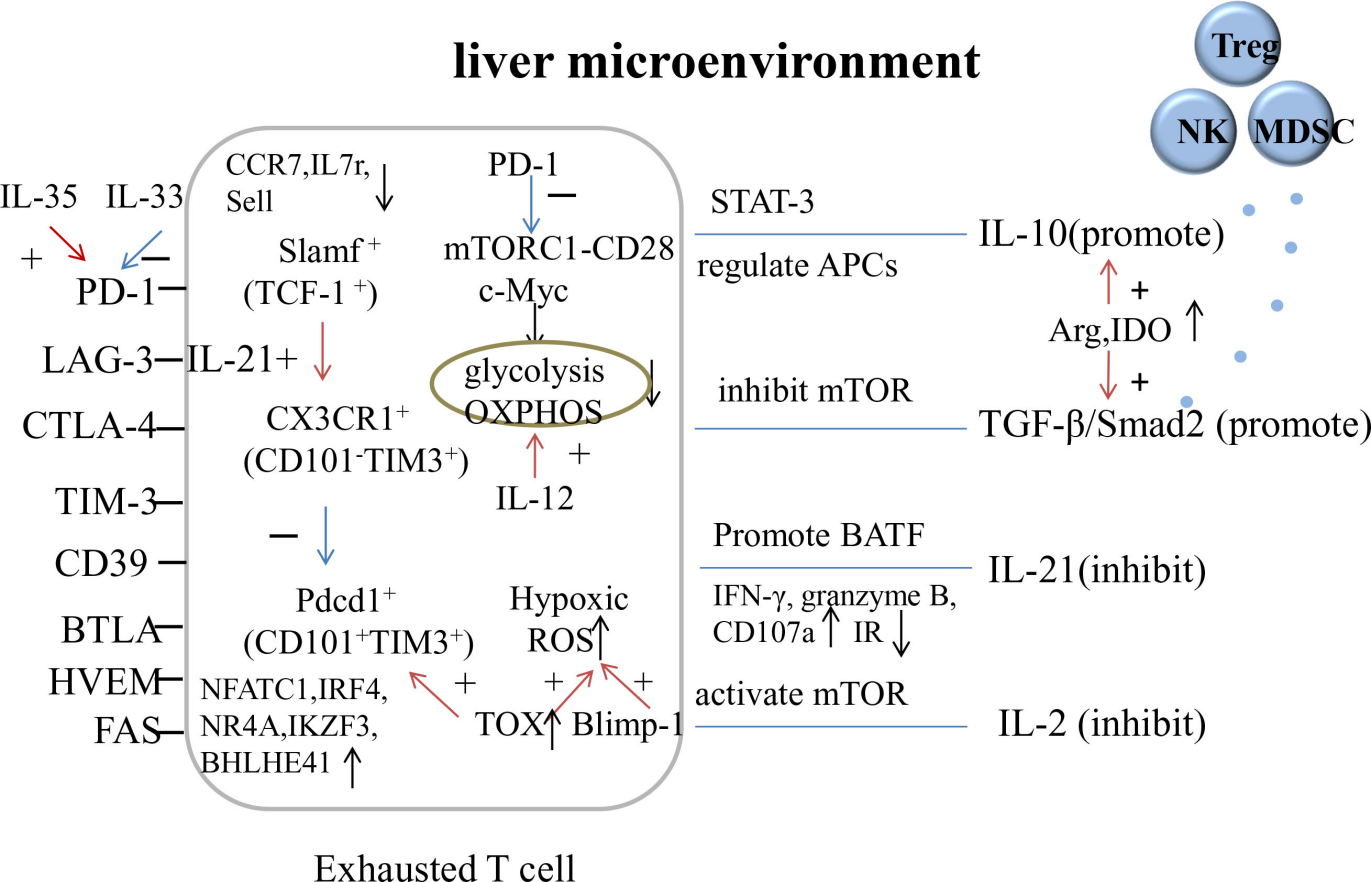
The mechanism of T cell exhaustion. OXPHOS, oxidative phosphorylation; Arg, arginase; IDO, indoleamine 2, 3-dioxygenase.

The deterioration of T cell function may be due to the hypoxic environment caused by increased β-oxidation in the liver, which leads to ROS accumulation in HBV-specific CD8^+^ T cells. Hypoxia-inducible factor (HIF) activation leads to metabolic reprogramming for adequate energy supply and cell proliferation but has little effect, while Blimp-1 prevents mitochondrial metabolic reprogramming (Barili et al., 2021). Hypoxia also increases IR expression and favors the accumulation and activation of MDSCs and Tregs. TOX also directly regulates CD8^+^ T cell metabolic genes, resulting in the increased expression of hypoxia response genes and decreased expression of genes regulating OXPHOS, mTOR signaling, IFN-α response, and DNA repair (Franco et al., 2020). The depletion of arginine and tryptophan, caused by the increased expression of Arg and IDO, affects the proliferation and metabolism of immune cells and regulates the secretion of IL-10 and TGF-β (Fisicaro et al., 2020). **Figure 2** shows the mechanism underlying T Cell exhaustion.

##### 3.1.1.2 Expression of inhibitory receptors

Inhibitory receptors comprise a group of molecules that act at immune synapses to inhibit T cell function, leading to T cell exhaustion. However, multiple IR may represent a recent or ongoing activation of CD8^+^ T cells that respond best to a given stimulus (Legat et al., 2013).

The IRs (PD-1, LAG-3, TIM-3, and CD39) on CD8^+^ T cells were increased in patients with CHB, and cell function was impaired (IFN-γ, perforin, and granzyme decreased). PD-1 and TIM-3 levels positively correlate with HBV DNA levels (Ye et al., 2017; Mohammadizad et al., 2019; Jiang et al., 2022). IL-35 promotes PD-1 expression via the JAK1/TYK2/STAT1/STAT4 pathway (Tang et al., 2021), while IL-33 induces proliferation of HBV-specific CD8^+^ T cells and decreases PD-1 expression. However, plasma levels of IL-33 are decreased in patients with CHB (Li et al., 2022a). LAG-3 is positively correlated with the severity of liver injury; an increase in LAG-3 inhibits the function of CD8^+^ T cells, reduces severe liver injury, slows HBV clearance, and prolongs HBV infection. In patients with negative HBeAg or HBc Ag < 5log^10^U/mL, IFN-α increased TLR2 on monocytes and did not increase PD-1 on CD8^+^T cells to promote virus clearance. In contrast, in patients with positive HBeAg or HBcAg > 5log^10^U/mL, IFN-α induced PD-1 on CD8^+^T cells and PD-L1 on monocytes and did not increase TLR2 on monocytes to mediate immunosuppression (Hakim et al., 2020; Huang et al., 2022a). TLR2 agonists and PD-L1 antagonists may inhibit HBV replication by inducing cytokine production and enhancing the cytotoxic effects of CD8^+^ T cells, particularly in patients with low HBV DNA and negative HBeAg (Ferrando-Martinez et al., 2019).

Th1 cells are significantly decreased in the peripheral blood of patients with CHB, and IRs (PD-1, LAG-3, TIM-3, and CTLA-4) are upregulated, which inhibit Th1 cell function (IFN-γ, IL-2, and TNF-α decrease). TNF-α-producing CD4^+^ T cells are the dominant cell population that contribute to liver damage while differentiating into IFN-γ-producing CD4^+^ T cells to favor clearance of the HBV (Wang et al., 2020). Blocking PD-L1 and LAG-3 prevents the production of Tregs and IL-10, and partially restores the function of CD4^+^ T cells (Park et al., 2016; Dong et al., 2019). A reduction in IL-12 levels induces a Th1/Th2 imbalance, resulting in a non-cytotoxic T cell response. Thus, IL-12 may be an interesting marker for predicting HBeAg seroconversion (Wiegand et al., 2019). In HBeAg-negative chronic asymptomatic HBV carriers, IRs on Th1, Th2, Th17, and Tregs (CD4^+^ T cell subsets) exhibit high expression (Buschow and Jansen, 2021; Cui et al., 2022). HBcAg increases Th17 cell proliferation (Zhang et al., 2011). Th17 cells secrete IL-17 and IL-22 in the presence of IL-6, IL-1β, IL-12, and IL-23 (Zhong et al., 2021). The mRNA expression of retinoic acid-related orphan receptor-γt (RORγt) is also significantly increased, exacerbating chronic liver inflammation and fibrosis. IL-17 activates MDCs and monocytes to release inflammatory cytokines and recruit neutrophils to the site of infection (Buschow and Jansen, 2021); the lower the methylation of the IL-17 promoter, the more severe the patient’s condition (Tian et al., 2019; Zhu et al., 2022). The Th17/Treg cell ratio increases and positively correlates with liver injury in patients with chronic HBV infection. The Th17/Treg cell balance may be a novel immunotherapy for patients with liver injury, as IL-35 regulates the ratio of Th17 and Treg cells (Liu et al., 2017a).

Both B and T lymphocyte attenuators (BTLA) and herpes virus entry mediators (HVEM) are co-signaling molecules. The combination of BTLA and HVEM produces signals that inhibit the activation and proliferation of T and B cells (Yu et al., 2019). The expression of BTLA and HVEM on the surface of CD4^+^ and CD8^+^ T cells is increased in patients with CHB and is positively correlated with liver injury. BTLA/HVEM signaling may play a regulatory role in T cell function through synchronous upregulation of the receptor/ligand. BTLA inhibitory signaling inhibits T cell proliferation, activation (CD25, CD69), and cytokine (IFN-γ, IL-2) production through IL-10 produced by BTLA^+^ Treg cells, leading to T cell exhaustion (Song et al., 2022). T cell suppression is restored by blocking BTLA signaling or neutralizing IL-10 (Wang et al., 2017b).

HBV-specific T cells induce early secretion of A3A and A3B by secreting IFN-γ and TNF-α in a synergistic fashion to generate deamination and subsequent cccDNA decay, reducing the level of cccDNA in a non-cytolytic manner and without direct contact. This is followed by the direct killing of infected hepatocytes in a cytolytic manner, with concomitant loss of cccDNA by cell division. However, T cell exhaustion and differences in gene polymorphisms of IFN-γ and TNF-α affect cccDNA clearance in patients with CHB. Because IFN-γ and TNF-α have severe side effects and cannot be used as drugs directly, therapeutic approaches to restore T cell response are used to achieve sustained control of HBV infection (Xia et al., 2016). IL-21-based gene and cell therapies have the potential to eliminate cccDNA by activating CD8^+^ T cells, accompanied by long-term protective memory (Shen et al., 2019). IFN-α is a commonly used anti-HBV drug, that mainly inhibits the transcription and replication of HBV. However, it cannot reduce cccDNA at HBV transcription and replication markers. High-dose IFN-α induces transient A3A and cccDNA degradation followed by rapid tolerance to IFN-α, which may account for the limited efficacy of IFN-α in treating CHB (Shen et al., 2018a). Lymphotoxin β receptor (LTβR) activation and IFN-α Treatment induce A3A and A3B deamination and apurinic/apyrimidinic (AP) site formation in cccDNA, and these sites are recognized by cellular AP endonucleases, leading to cccDNA lysis. LTβR agonists are effective at low doses without any toxicity observed *in vitro* and *in vivo*. Drugs that induce A3A and/or A3B activity, such as LTβR agonists or adoptive T cell therapies, should be used in combination with nucleotide analogs to avoid supplementation with degradative cccDNA (Lucifora et al., 2014). TCR-reprogrammed resting T cells selectively activate LTβR and APOBEC3 in a temporary manner upon antigen recognition and suppress HBV replication without lysis and triggering inflammatory events, which may be a beneficial therapeutic strategy for CHB (Koh et al., 2018).

#### 3.1.2 Other T cell subsets

CD4^+^CXCR5^+^ TFH cells are a subset of effector T cells that express CD40L and promote T cell-dependent B cell (B cell activation and antibody type switching) and GC responses (Qi, 2016). CXCL13, a ligand of CXCR5, determines the homing and motility of CXCR5+ cells, and promotes the migration of B and TFH cells to lymphoid follicles and GC. TFH cells secrete IL-21 to promote B cell maturation and antibody production. High frequency of TFH cells promotes HBeAg seroconversion in patients with CHB. PD-1 controls the tissue localization and function of TFH cells in a costimulus-dependent and independent manner (Shi et al., 2018b). According to CCR7 and PD-1 on the surface of TFH cells, there are two major subsets of circulating TFH cells. The CCR7^-^PD-1^+^ subset exhibits a TFH precursor phenotype and active TFH differentiation. In contrast, the CCR7^+^PD-1^−^ subset exhibited resting cell characteristics. The proportion of CCR7^-^PD-1^+^ TFH cells in the peripheral blood of patients with CHB is higher than that in healthy individuals, and is positively correlated with ALT levels, which are involved in the immune response to chronic HBV infection (Huang et al., 2018). Moreover, HBV may impair the function of TFH cells by enhancing Treg activity (Liu et al., 2022d).

CD8^+^CXCR5^+^ T cells are follicular cytotoxic T (TFC) cells. The transcription factor E2A activates CXCR5 transcription, whereas Blimp1 inhibits this process. They form a transcriptional circuit with Bcl6 and TCF-1 to guide TFC development (He et al., 2016). The deletion of the E2A regulator ID3 resulted in increased CXCR5 expression. Bcl6 is a classical transcription factor essential for GC formation. Therefore, TFC have follicular helper features but reduced cytotoxic function due to Bcl6 expression (Shen et al., 2018b). TFC cells express high levels of IR (PD-1, CTLA-4, and TIM-3) and activation markers CD38 and CD69. Therefore, IR expression not only marks cell dysfunction but also correlates with activation and differentiation (Legat et al., 2013). Serum TFC were partially exhausted in patients with CHB but could produce high levels of IFN-γ and IL-21 and have antiviral ability. RNA sequencing showed that CXCR5^+^ cells had high transcriptional levels of molecules encoding the TNF and IFN pathways, but low transcriptional levels of cytotoxic effector molecules (granzyme, granulysin, and perforin), which may be related to the decrease in Blimp1 expression. This indicates that the antiviral effect on cells was mainly mediated by cytokine secretion rather than cytolysis. CD62L, CCR7, and CD45RO are highly expressed in cells that show characteristics of memory precursor cells and memory potential. CXCL13mRNA in the liver is significantly increased in patients with CHB and is induced by TLR3 ligand stimulation, which promotes the recruitment of CXCR5^+^ lymphocytes to the liver. Intrahepatic TFC have a stronger antiviral effect than circulating TFC, which is beneficial for controlling HBV infection. TFC cells promote immunoglobulin production and support B cell responses. CXCL13 is associated with viral control and the CXCR5^+^ subset with proliferative capacity is a functional subset of the most dysfunctional CD8^+^T cells. This provides potential targets for the immunotherapeutic of CHB (Li et al., 2015b; Im et al., 2016; Liu et al., 2017b).

CD8^+^ tissue-resident memory T (TRM) cells reside in the liver mainly as CD69^+^CD103^+^ subsets (Bolte and Rehermann, 2017), lack lymphatic homing molecules CD62L and CCR7, and downregulate S1PR1 and KLF2 expression (Han and Shin, 2020). HBV-specific TRM cells control viral replication and aid in the functional treatment of patients with CHB (Li et al., 2022c). These cells enhance basal autophagy to adapt to the highly damaged mitochondrial environment of the liver, and are better able to proliferate, mediate cytotoxic effects, and produce cytokines. Blocking autophagy leads to the accumulation of depolarized mitochondria, which is characteristic of Tex cells. Thus, the upregulation of autophagy enables CD8^+^ T cells to counter mitochondrial depolarization and acquire tissue residency (Swadling et al., 2020).

### 3.2 Humoral immunity

The humoral immune response involves the differentiation of pathogen-specific B cells into plasma cells that produce antibodies to neutralize the infection. HBsAb can bind to HBsAg to induce antibody-dependent cytotoxicity and phagocytosis to kill and phagocytose HBV-infected cells. B cell-mediated humoral immunity is often overlooked in chronic HBV infection. However, treatment with B cell-depleting agents such as an anti-CD20 monoclonal antibody (rituximab) can reactivate HBV replication, leading to increased liver inflammation and fatal outcomes, even in patients with a resolved infection (Li et al., 2021; Vanwolleghem et al., 2022). As a member of the APOBEC family, activation-induced cytidine deaminase (AID) is specifically expressed in activated B cells and is necessary for antibody class switching and somatic hypermutation, which produces high-affinity antibodies. TGF-β1 can target cccDNA via AID and trigger uracil-DNA glycosylase/AP to mediate cccDNA deamination and degradation. This is the methods for the potential elimination of cccDNA (Qiao et al., 2016).

B cells in patients with CHB are over-activated, with high expression of activation markers (CD69, CD71, CD80, and CD86) and IR (PD-1, FcyRIIB, and FcRL5) and low expression of CD21 and CD27, which decrease proliferation ability and increase apoptosis, causing HBsAg-specific B cell function defects and reducing the production of IL-6 and TNF (Burton et al., 2018; Salimzadeh et al., 2018; Tian et al., 2018). The humoral immune response is primarily against HBcAg, which activates B cell maturation without the need for helper T cells. HBcAg-specific B cells are more common than HBsAg-specific B cells and are associated with the natural history of CHB. HBcAg-induced B cell responses are high and significantly decreased during antiviral therapy (Vanwolleghem et al., 2020). HBcAb levels at the end of treatment can predict clinical recurrence in patients with CHB infection who discontinued treatment (Chi et al., 2019). HBcAg-specific B cells mature into plasma cells *in vitro*, presenting an IgG^+^ memory B cell phenotype, whereas HBsAg-specific B cells do not mature into plasma cells *in vitro* and show a major IgM+ phenotype. The expression of CD31/PECAM1 and NFTAC1 (upregulated in HBsAg-specific B cells), or IL-6R and CXCR3 (upregulated in HBcAg-specific B cells), which are involved in the regulation of B cell development, confirmed the defective maturation of HBsAg-specific B cells and increased the memory function of HBcAg-specific B cells. However, HBcAg-specific and HBsAg-specific B cells highly express genes for cross-presenting DC recruitment (XCL1 and CD40LG) and innate immunity (MYD88, IFNA1/13, IFNa2, and IFNB1), despite their phenotypic and functional differences (Le Bert et al., 2020b). The induction of HBsAg-specific B cells is a potential approach to immunotherapy (Xu et al., 2015).

B cell functions differ at different stages of chronic HBV infection, and the activation status of B cells is high in the immune active and inactive carrier stages, with upregulation of genes encoding the activation markers CD69 and CD83 and downregulation of genes encoding IRs, consistent with HBeAg seroconversion. The upregulation of several IFN-stimulated genes (ISGs) indicates B cell functions other than antibody production during the immune active stage. However, the immune tolerance and reactivation stages showed no signs of B cell activation, suggesting that these two stages were driven by other immune cells or viral factors. Immune signaling pathways related to activation-related genes (CD69 and CD83), chemokine receptors (CCR1, CCR2, CCR5, CCR7, CX3CR1, and CXCR2), and ISG were significantly upregulated in intrahepatic B cells compared to those in peripheral B cells. The liver environment may influence B cells by interacting with the signals from HBV-infected hepatocytes, other parenchymal cells, and intrahepatic immune cells (Van Hees et al., 2021).

B cells as APCs can present HBV-associated antigens to CD4^+^ T cells via MHC-II molecules. However, HBsAg- and HBcAg-specific B cells can cross-present HBsAg and HBcAg to CTL through MHC-I molecules, resulting in the death of HBsAg- and HBcAg-specific B cells (Cai and Yin, 2020) and impeding the production of HBsAb, thereby promoting persistent HBV infection. The levels of costimulatory molecules on B cells are reduced, and their antigen presentation function is potentially impaired. Reversal of B cell hyperactivation and function is associated with HBsAg seroconversion in patients with CHB. The underlying mechanisms of IFN-α treatment are associated with reinforced connections between B and T cells mediated by antigen presentation and costimulatory interactions (Zhong et al., 2022).

## 4 Inhibitory cells in immune response

### 4.1 Treg

CD4^+^CD25^+^ Tregs are a subset of CD4^+^ cells that can be classified into natural and induced Tregs. Induced Tregs are usually induced by inflammatory and disease processes and favor the establishment of chronic infections. Tregs inhibit immune function, induce immune tolerance, and are associated with the PI3K/Akt/mTOR pathway. Most Tregs in healthy individuals are in the resting state. Resting Treg cell activation, ID3 (DNA-binding inhibitor) expression, and inhibitory ability are gradually enhanced during persistent HBV infection (Liu et al., 2021a), which downregulates the antiviral activity of effector T cells and recruits innate immune cells to the infected liver, leading to incomplete viral clearance. IL-1β upregulates ID3 expression and inhibitory cytokines such as IL-10, IL-35, and TGF-β, are key mediators of Treg function. IL-10 inhibits host anti-HBV activity, leading to continuous replication and expression of HBV. Increased IL-10 levels are correlated with HBV DNA and liver inflammation (Trehanpati and Vyas, 2017). IL-35 increased the proliferation of Tregs and inhibited the proliferation and effector functions of T cells in patients with CHB. HLA-DQ enhances the suppressive function of Tregs (Zeng et al., 2022). Decreased PD-1 and GITR expression weakens the immunosuppressive capacity of Tregs and enhances the antiviral function of effector T cells (Liu et al., 2022a). Treatment by reducing the suppressive effect of Treg cells may improve immune status (Pang et al., 2020).

Tregs inhibit IFN-γ-producing CD8^+^ T cells and directly inhibit their antiviral functions. The activation and cytotoxicity of NK cells are directly inhibited through the interaction of mTGF-β and OX40/OX40L (Chen et al., 2016). CTLA-4, IL-10, and TGF-β inhibit DC maturation, and the interaction between Tregs and DCs reduces the activation of effector T cells by DC. It can also induce the death of antigen-presenting DC in the lymph nodes in a perforin-dependent manner. HBsAg-specific Tregs mediate TFH-dependent HBsAb dysregulation by limiting the differentiation of HBsAg-specific TFH cells, resulting in insufficient HBsAb against a large number of HBV-infected hepatocytes, as HBV persists throughout the body. Removing Tregs or blocking CTLA-4- and HBsAg-specific TFH cells in patients with CHB recovers their ability to clear HBV, which can be used as a target for CHB treatment (Wang et al., 2018b).

Follicular regulatory T (TFR) cells, which express both Treg and TFH markers, limit excessive GC responses by inhibiting TFH and B cells. Glycoprotein A repetitions (GARP) are predominantly upregulated during Treg activation. Tcf-1 promotes self-renewal of CD8^+^ T cells and suppresses effector CD8^+^ T cells. Increased TFR cells and decreased IL-21 levels promote the differentiation of Breg cells in patients with CHB and suppress plasmablast differentiation and IFN-γ from CD8^+^ T cells (Wang et al., 2018a). The expression of GARP and Tcf-1 in Tregs and TFR cells is upregulated in patients with CHB, and Tcf-1, CD62L, and GARP-classified Treg and TFR subsets are altered (Bahabayi et al., 2022).

### 4.2 Breg

Similar to Tregs, the number of Bregs in patients with CHB is high and are mainly immature/transition cells. The immune active stage is the highest and is negatively correlated with IL-17 and IFN-γ secreted by CD4^+^ T cells (Th1 and Th17 cells) and CD8^+^ cells (Gong et al., 2015; Jin et al., 2021) but positively correlated with IL-4-producing T cells (Th2 cells). Bregs can increase the number of Tregs and secrete inhibitory cytokines. Bregs dysregulate T cell function through IL-10, TGF-β, IL-35, and cell-to-cell contact. IL-35 levels correlate with the deterioration of liver cirrhosis. TLR9 levels decreased, and TLR2 levels increased in the Bregs of patients with CHB. Progression of inflammation may promote the elevation of Bregs to prevent excessive immune responses, but may also contribute to the persistence of HBV (Xiang and Xie, 2015; Liu et al., 2016; Wang et al., 2017a; Liu et al., 2021c).

### 4.3 MDSC

HBsAg upregulates CCR9 expression in M-MDSCs by activating ERK1/2 and IL-6 in patients with CHB. CCL25 is the only CCR9 ligand secreted by the thymus and the interaction between CCL25 and CCR9 mediates the thymic homing of M-MDSCs and transfers HBsAg to the thymic medulla. This leads to antigen cross-presentation and promotes the death of HBsAg-specific CD8^+^ cells via NADPH oxidase, leading to immune tolerance. HBeAg also promotes the production of M-MDSCs dependent on IL-6 and IL-1β. The synergistic effect of inhibitory molecules (Arg1, iNOS, PD-L1, and CTLA-4) and increased IL-4, TNF-α, and IL-1β enhance the inhibitory effect of M-MDSCs and promote the expansion of FOXP3^+^ Tregs and IL-10 ^+^ Tr1 cells. Furthermore, it impairs HBV-specific T cell functions (proliferation, activation, and degranulation) through TGF-β and IL-10-dependent signaling pathways and IDO and weakens effective antiviral response (Huang et al., 2014; Yang et al., 2019; Pal et al., 2022). The presence of M-MDSCs impairs CCR5-guided HBV-specific T cell motility, and low levels of CCR5 lead to the ineffective recruitment of HBV-specific T cells to the liver. Patients with fewer M-MDSCs are more likely to achieve a functional cure (Zhang et al., 2023). Simultaneously, G-MDSCs express arginase and liver-homing chemokine receptors that increase and accumulate in the liver, and HSCs support their expansion, effectively inhibiting T cells in a partially arginase-dependent manner (Pallett et al., 2015). One year of tenofovir treatment failed to restore the phenotypic and functional characteristics of MDSCs and Tregs, and reduced the HBsAg load, indicating that there is still an immunosuppressive cascade (Pal et al., 2019), which may be a key risk factor for advanced liver diseases, such as HCC. CCR9 neutralization can block the thymic homing of M-MDSCs and the deletion of HBsAg-specific CD8^+^ cells by M-MDSCs, thus interrupting HBV persistence. Selective interference or neutralization of CCR9 in M-MDSCs may be a novel treatment strategy (Fang et al., 2022).

### 4.4 Natural killer-reg

Monocytes mediate NK-reg production during cell-to-cell contact via PD-L1/PD-1 and HLA-E/CD94. They express TGF-β, IL-10, and low levels of IL-12, IL-18, and T-bet, and inhibit the activation of T cells in an IL-10-dependent manner. These results indicate that HBV-induced NK-reg cells directly inhibit the antiviral effect of NK cells and downregulate the T cell immune response. Blocking PD-L1/PD-1 and HLA-E/CD94 significantly inhibits the expression of IL-10 and increases IFN-γ levels in NK cells. Therefore, altering the activity of NK-reg cells is a potential therapeutic strategy (Li et al., 2018).

## 5 Conclusion

Chronic HBV infection can cause a series of immune tolerance cascades. The number and function of antigen-presenting cells such as DCs and macrophages in the innate immune response and inflammatory cytokines are reduced, leading to delayed or weak adaptive immune responses. Natural killer cells and exhausted T cell-mediated cytotoxicity cannot effectively kill HBV-infected cells. Suppressor cells and cytokines can lead to persistent and chronic HBV infections. All the above-mentioned abnormal immune mechanisms can be used as potential therapeutic targets for chronic HBV infection to improve the immune response. However, further exploration and clinical trials are still needed to ensure that individualized therapies targeting host immunity alone or in combination with antiviral drugs can achieve viral clearance and the goal of a functional cure.

## Author contributions

PZ wrote the review, PZ and YD searched comprehensive literature.YD and QW revised the review. All authors contributed to the article and approved the submitted version. 
